# Assessment of Knowledge and Awareness Among Nurses of a Tertiary Care Hospital Regarding the Management of Acute Poisoning: A Cross-Sectional Study

**DOI:** 10.7759/cureus.36781

**Published:** 2023-03-28

**Authors:** Kusum Kumari, Mary S Toppo, Md S Alam

**Affiliations:** 1 Pharmacology, Rajendra Institute of Medical Sciences, Ranchi, IND

**Keywords:** acute poisoning, regression, nurses, knowledge, awareness, poisoning

## Abstract

Background: Poisoning is a major health issue worldwide. Nurses are frequently the first healthcare professionals to come in contact with patients with acute poisoning. So, this study was conducted to assess the knowledge and awareness regarding the management of acute poisoning among nurses. In case of insufficient knowledge, they could be trained enough to manage any case of acute poisoning for life-saving purposes.

Methodology: This was a cross-sectional analytical study conducted among 212 nurses at Rajendra Institute of Medical Sciences, Ranchi, Jharkhand from July 2021 to December 2021. Data were collected using the structured questionnaire based on a previous study and regression analysis was used for determining the association between their overall knowledge score and independent variables like age, education, experience, and training status.

Results: More than one-third (43.8%) of the study participants had an unsatisfactory level of knowledge, which might be because of a lack of proper training and experience, but still, 42.4% and 13.6% of the study participants had satisfactory and good knowledge, respectively. Moreover, more than one-third (45.2%) of the study participants had inefficient skills, while 48.1% and 6.6% of nurses had satisfactory and good skills, respectively, in the management of acute poisoning. The three independent variables (training, experience, and education) showed a 53.1% association with total scores in bivariate regression analysis while no significant association was seen between age and score in multivariate analysis.

Conclusions: About half of the nurses showed unsatisfactory knowledge, so they need improvement and they should be trained regarding this to reduce mortality among acute poisoning cases.

## Introduction

Poison is any substance that when introduced into our body or when it comes in contact with the body parts, either accidentally or intentionally, causes some harmful effects or even death. Any substance can act as poison if taken at a higher dose [1]. So, it is the quantity that makes any substance act like a poison. The pattern of acute poisoning varies from place to place according to the availability of poisoning agents. In a study conducted in South Africa [2], the percentage of accidental poisoning was 59%. Agricultural pesticides such as organophosphorus (OP) compounds, organochlorine, zinc phosphide, and aluminum phosphide are commonly used poisoning agents in Asian countries. OP compounds are used as suicidal or accidental poisonings in rural areas of developing countries [3], while misuse of the drugs such as opioids, benzodiazepines, and tranquilizers is commonly seen in developed countries. The number of cases of suicidal poisoning has risen not only in Sri Lanka [4], but also in England and Wales [5]. Poisoning is more common in males all over India [6]. It has become a leading cause of increased morbidity and mortality [7]. In developed countries, the mortality rate from poisoning is only 1-2%, whereas, in developing countries like India, it varies from 15% to 30%. Poisoning is the fourth most common cause of mortality in India [6]. Often it has been found that the majority of the victims of poisoning belong to lower socioeconomic countries. Due to the lack of updated information and poor accessibility of healthcare facilities, our country is facing a lot of problems leading to more fatalities. An online survey was conducted in India between September 2015 and May 2016 to understand the trend of the presentation of poisoned cases [8]. In India, generally emergency physicians provide the initial evaluation and management of any poisoned case.

Rajendra Institute of Medical Sciences (RIMS), Ranchi is a tertiary care hospital in Jharkhand with well-qualified physicians. But, because of crowded emergency and outpatient consultations, there is often a delay in attending the emergency cases like poisoning that may lead to fatality. So, nurses may play a very important role in such cases. For this, they need sufficient knowledge. Very few studies are available in Jharkhand regarding this. Therefore, this study was conducted to assess the knowledge and awareness regarding the management of acute poisoning among nurses, so that the participants with unsatisfactory knowledge could be trained to manage such cases more efficiently.

## Materials and methods

Objective of the study

To assess the knowledge and awareness in the management of acute poisoning among nurses and to find out the association of different independent variables with total scores.

This was a cross-sectional analytical study conducted among 212 nurses in RIMS, Ranchi, Jharkhand from July 2021 to December 2021. Ethical permission was taken from the Institutional Ethical Committee (IEC) RIMS, Ranchi (IEC registration no.: ECR/769/INST/JH/2015/RR-18; approval no.: 82; dated: 22.10.2020). The research was carried out in the Department of Pharmacology and Therapeutics, RIMS, Ranchi, Jharkhand.

The sample size was calculated as 210 by assuming the power of the study to be 80%, with a 5% margin of error and adding 10% for the dropout rate.

Sampling and data collection procedure

After taking proper informed consent from study participants, we included those nurses who were readily accessible to the hospital and had given informed consent to participate in the study. Exclusion criteria were nurses doing basic training courses, nurses who were on leave, and those who were not directly involved in immediate patient management like working in the radiology department. The structured questionnaire was framed based on one hospital-based cross-sectional study conducted among nurses working in Dessie Referral Hospital, Amhara region, Ethiopia [9]. The questionnaire was designed in three sections (A, B, and C). Section A included sociodemographic characteristics of the study participants, their educational status, experience, and training status related to the management of poisoning. Section B included seven questions regarding general knowledge of poisoning. Each correct answer was given a score of 1, while the incorrect answer was rated as 0. Based on the appropriate response to the given 13 items in Section B, a score of 0-13 was assigned to each participant. Section C of the questionnaire included 10 questions related to the initial management of poisoning and 1 score was given for each correct answer and 0 for the wrong answer (Appendix). Based on responses to the given 10 items in Section C, a score of 0-10 was given to each participant. Their total score varies from 0 to 23. A score of more than 75% means good knowledge, between 50% and 75% means acceptable knowledge, and less than 50% means insufficient knowledge or poor score.

Statistical analysis

The data were entered into an Excel sheet (Microsoft Corporation, Redmond, WA) and analyzed in the form of mean and percentage. A p-value of less than 0.05 was considered to be significant. Both bivariate and multivariate regression analyses were done using SPSS version 22 software (IBM Corp., Armonk, NY) for determining the association between the independent variables (training, age, experience, and education) and the dependent variable (total score).

## Results

Out of 225 study participants, a total of 212 nurses completed the questionnaire with a response rate of 94.22%. Sociodemographic data along with experience and training status on acute poisoning are shown in Table 1. The mean age of the nurses was 48.14 ± 6.67 years. About 32% of the nurses had previous experience in managing a case of acute poisoning. On the other hand, about 25% of nurses had already taken training for the management of acute poisoning cases.

**Table 1:** Sociodemographic data, experience, and training status of the study participants

Characteristics		Frequency (n = 212)	Percentage
Age (years)	25-29	5	2.3%
	30-34	3	1.4%
	35-39	7	3.3%
	40-44	36	16.9%
	45-49	65	30.6%
	50-54	44	20.7%
	55-60	52	24.5%
Experience in poisoning management	No	145	68.3%
	Yes	67	31.6%
Training in poisoning management	No	158	74.5%
	Yes	54	25.4%

**Figure 1:**
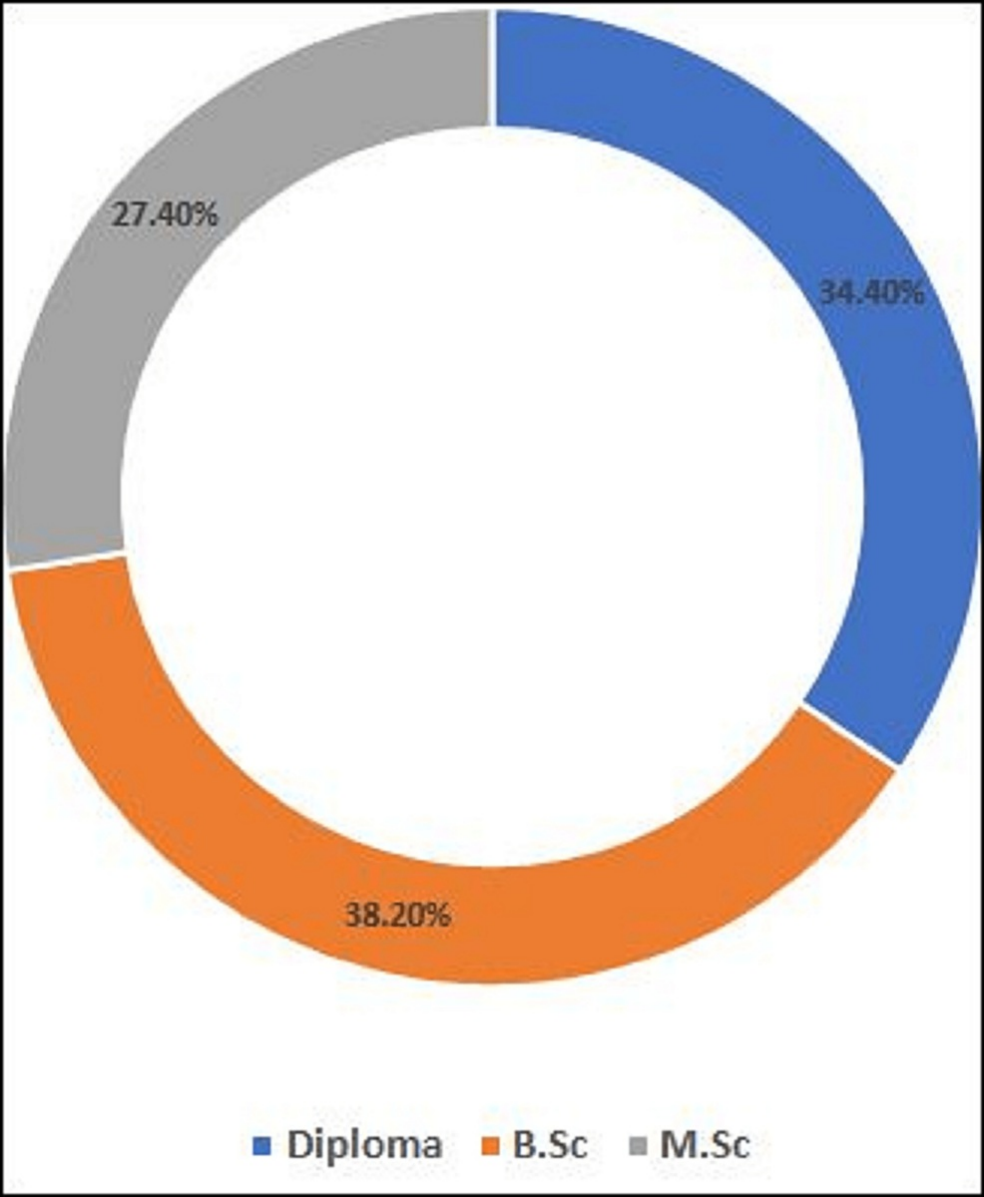
Educational status of study participants

The education status of the nurses has been shown in Figure 1.

Nurses’ response on general knowledge and initial management of acute poisoning has been shown in Table 2 in the form of scores obtained. In section B, 43.8% of nurses got a score of less than 50%, whereas 42.4% got a score between 50% and 75%, and only 13.6% of nurses got a score of more than 75%. Similarly, in section C, 45.2% of the nurses obtained a score of less than 50%, 48.1% got a score between 50% and 75%, and only 6.6% of the study participants obtained a score of more than 75%.

**Table 2:** Nurses' response on general knowledge and initial management of acute poisoning

Variables	Scores	Frequency (n = 212)	Percentage (%)
General knowledge of poisoning	3-6	93	43.8
	7-10	90	42.4
	11-13	29	13.6
Knowledge of initial management of acute poisoning	2-4	96	45.2
	5-7	102	48.1
	8-10	14	6.6

**Figure 2:**
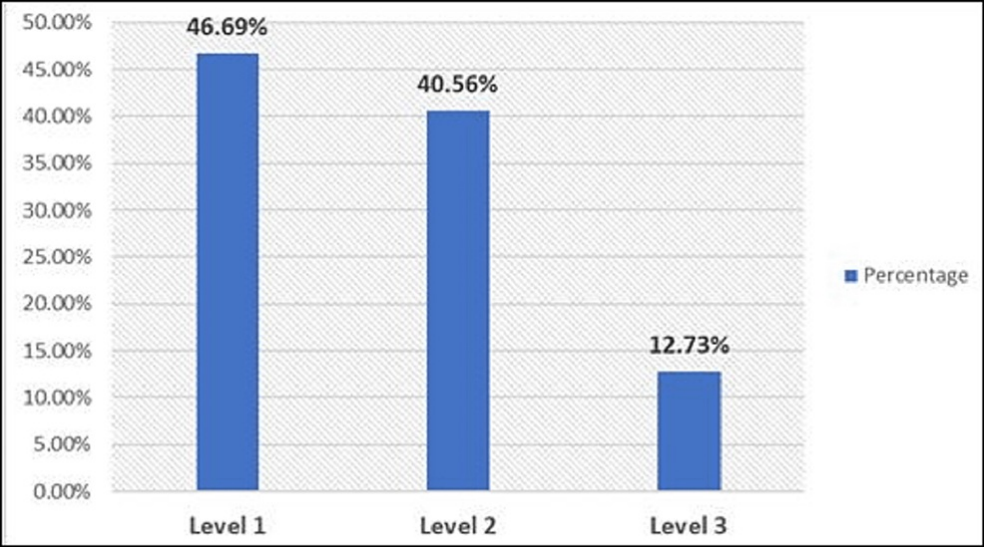
Nurses' response on overall knowledge of acute poisoning

Out of the total 23 scores, the total mean score obtained was 12.98 ± 4.32. Total scores have been shown in Figure 2. Total scores were divided into three levels. Scores below 50% were labeled as level 1, between 50% and 75% as level 2, and more than 75% as level 3. So, about 47% of the participants secured less than 50% of the total score and only 12.73% obtained more than 75%.

To investigate the association between dependent and independent variables, linear regression analysis (both bivariate and multivariate analyses) was done. The result of the bivariate regression analysis has been shown in Table 3. Four hypotheses (H_1_, H_2_, H_3_, and H_4_) were considered in which the dependent variable was the total score obtained by the nurses and the independent variables were training in H_1_, age in H_2_, experience in H_3_, and education in H_4_. Our hypothesis was the following: there is a significant impact of independent variables on the dependent variable (total score). In bivariate analysis, all four hypotheses were supported (p < 0.05), although age showed a negative association (β = -0.178).

**Table 3:** Results of bivariate analysis

Hypothesis value	Regression weights	β-coefficient	F	P
H_1_ <0.001	Training - total score	0.661	0.437	163.165
H_2_ 0.009	Age - total score	-0.178	0.032	6.882
H_3_ <0.001	Experience - total score	0.707	0.501	210.456
H_4_ <0.001	Education - total score	0.354	0.125	30.055

In multivariate analysis, only H_1_, H_3_, and H_4_ hypotheses were supported and the H_2_ hypothesis was not supported (p = 0.178). R^2^ = 0.531 indicates that the three independent variables (training, experience, and education) have a 53.1% association with the sores. The result of the multivariate analysis has been shown in Table 4.

**Table 4:** Results of multivariate analysis R = 0.729; R^2^ = 0.531.

Hypothesis	Regression weights	β-coefficient	t	P-value
H_1_	Training - total score	0.19	2.03	0.044
H_2_	Age - total score	-0.066	-1.352	0.178
H_3_	Experience - total score	0.496	5.247	<0.001
H_4_	Education - total score	0.120	2.344	0.020

## Discussion

Poisoning is a major health issue worldwide. Acute poisoning increases the number of hospitalization as well as the number of emergency cases. It is the cause of more than 3 million illnesses throughout the world. Acute poisoning is a very important medical emergency [10]. It requires teamwork, so, for better management of such cases, all team members should have sufficient knowledge as well as skills. In acute poisoning cases, the role of nurses becomes very important. Less knowledge of nurses may be the cause of medication errors also [11]. So, the motive of this study was to assess the knowledge and awareness regarding acute poisoning among the nurses working in RIMS, Ranchi.

According to this study, most of the study participants (47.6%) were in the age group of 40-49 years with a mean age of 48.14 ± 6.66 years. Regarding their education details, most of the nurses (38.2%) were having a Bachelor of Science degree. This might be because the study was conducted in a tertiary care teaching institute where a nursing college is also functional for the deployment of most of the nurses in the hospital. Most of the nurses (43.8%) had an unsatisfactory level of knowledge about acute poisoning. This is similar to the findings reported in the studies done in Kenya [12] and Ethiopia [13]. However, 42.4% of the study participants had an acceptable level of knowledge and only 13.6% had good knowledge. This might be because of their different education levels and is supported by our finding of the least percentage of nurses with a Master of Science degree (27.4%). As far as the initial management of acute poisoning is concerned, most of the nurses (48.1%) had an acceptable level of knowledge and 45.2% had insufficient knowledge. Whereas, only 6.6% of nurses had good knowledge. This variation in the level of knowledge might be because of differences in experience and training status among nurses [14]. Our study supports this evidence as only 25% of the nurses are trained and 32% are experienced in acute poisoning management. In one study conducted in Japan, a great difference was found between actual practice and awareness among stroke care unit nurses [15]. In an online survey reported in Singapore, it was found that nurses with higher education or experience in an emergency got more knowledge scores [16]. A similar result was found in a cross-sectional study in Ethiopia [17]. In China, the implementation of an e-learning intervention approach regarding palliative care became very helpful in enhancing the knowledge and attitude among nurses [18].

Regarding overall total scores, in sections B and C, 46.69% of the nurses got poor scores while 40.56% got fair scores and only 12.73% got good scores. It clearly depicts that the nurses have insufficient knowledge.

To investigate the association between dependent and independent variables, linear regression analysis (both bivariate and multivariate analysis) was done. The results of bivariate analysis depicted that training (β-coefficient = 0.661), experience (β-coefficient = 0.707), and education (β-coefficient = 0.354) have significant (p < 0.001) and positive association, whereas, age has a negative association (β-coefficient = -0.178) but is significant (p = 0.009). It clearly indicates that experience has a maximum association (70.7%) with the scores followed by training, which shows a 66% association. A negative association with age may be due to the gradual loss of memory as age advances. It is quite obvious that only experienced and trained nurses can do better management of a poisoned case. Since nurses come as first contact persons for the patient as well as relatives and they accompany them physically, mentally, and spiritually [19], they should be trained enough to get sufficient knowledge. Our finding is similar to the finding of Saad et al. [20]. The result of multivariate analysis demonstrated that age does not have a significant association with the scores. But the other three independent variables (training, experience, and education) have a 53.1% association with the scores. So when employing nurses, experience, as well as training status, becomes very important besides education.

Limitations of the study

The study was conducted only to assess the knowledge and awareness among nurses of a tertiary care hospital in the management of acute poisoning. However, their attitude as well as practices in the management of acute poisoning were not taken into consideration.

Novelty

For the first time we assessed the knowledge of nurses regarding the management of acute poisoning in Jharkhand and we also did the regression analysis for computing the strength of association between the nurses' performance in the form of scores and their education, training, and experience level. Also, regression analyses, both bivariate and multivariate, were done to determine the association between the dependent variables and independent variables.

## Conclusions

Nurses are the first contact persons for acute poisoning subjects as well as their relatives. This study describes the education details, experience level, and training status of the nurses working in RIMS, Ranchi. About half of the nurses have unsatisfactory knowledge regarding poisoning management and only 12.73% have good knowledge, which is not acceptable. Since nurses play a very important role in acute poisoning case management, they need training to enhance their knowledge and for better management of poisoned cases.

Experience and training show a stronger association than education with management skills, so medical institutions should conduct frequent training programs to enhance medical professional skills. We all know that experience is very important in the medical field. Knowledge can be gained by self-study via books or attending training but skills can only be developed through experience, which is very essential while managing a poisoned case or any other emergency. So, they should be trained and educated via conducting different training programs from time to time to enhance their management capacity, which will be helpful in reducing mortality among acute poisoned cases.

## Appendices

Questionnaire

**Table 5:** Section A: age details, education, experience, and training status

Question	Response
Year of birth/date of birth	
Educational qualification - B.Sc/diploma/M.Sc	
Experience in an emergency - yes/no	
Have you managed any cases of acute poisoning - yes/no	
Any training courses for poisoning management - yes/no	

**Table 6:** Section B: questions regarding general knowledge on acute poisoning

S. No.	Question	Response
1	Poison is any substance capable of causing damage to the body parts - TRUE/FALSE	
2	Dose and time of ingestion are not very necessary while managing a poisoning case - TRUE/FALSE	
3	Treatment of poison is more important than the treatment of the patient - TRUE/FALSE	
4	Pesticide poisoning is more common in developing countries - TRUE/FALSE	
5	Personal details of the poisoned patients like address and identification marks are not important - TRUE/FALSE	
6	The cause of poisoning in the emergency department, according to motive and nature of use, can be classified as follows: I. Deliberate poisoning - TRUE/FALSE	
	II. Accidental poisoning - TRUE/FALSE	
	III. Homicidal poisoning - TRUE/FALSE	
	IV. Euthanasia poisoning - TRUE/FALSE	
7	Alimentary signs and symptoms of acute poisoning during the early stages are: I. Dry mouth, abdominal pain, and salivation - TRUE/FALSE	
	II. Nausea, vomiting, hallucinations and convulsions - TRUE/FALSE	
	III. Coughing, cyanosis, hyperventilation, and salivation - TRUE/FALSE	
	IV. Tachycardia, hypotension, diarrhea, and breathlessness - TRUE/FALSE	

**Table 7:** Section C: questions related to the initial management of acute poisoning

Question	Response
1. In severe acute poisoning, maintaining adequate airway, respiration, and circulation is always a priority - TRUE/FALSE	
2. In case of organophosphate poisoning, atropine should not be administered in any circumstance - TRUE/FALSE	
3. Nearly all poisoning encountered in the accident and emergency departments have their specific antidote - TRUE/FALSE	
4. The decision to perform gastrointestinal (GI) decontamination should be based upon the specific poison(s) ingested, time from ingestion to presentation, and the predicted severity of the poison - TRUE/FALSE	
5. Activated charcoal can increase the absorption of a wide range of poisons from the gastrointestinal tract to the entire human system - TRUE/FALSE	
6. Gastric lavage is indicated for patients who have ingested kerosene or corrosive substances within an hour of presentation - TRUE/FALSE	
7. The effectiveness of gastric lavage increases as the time between ingestion and treatment increases - TRUE/FALSE	
8. The volume of lavage fluid aspirated should approximate to the amount of fluid given - TRUE/FALSE	
9. Patients presenting following ingestion of controlled/slow-released substances may benefit from decontamination even after a longer delay - TRUE/FALSE	
